# Oxygen reactions on Pt{*hkl*} in a non-aqueous Na^+^ electrolyte: site selective stabilisation of a sodium peroxy species†
†Electronic supplementary information (ESI) available. See DOI: 10.1039/c8sc05489d


**DOI:** 10.1039/c8sc05489d

**Published:** 2019-01-17

**Authors:** Thomas A. Galloway, Jin-Chao Dong, Jian-Feng Li, Gary Attard, Laurence J. Hardwick

**Affiliations:** a Stephenson Institute for Renewable Energy , Department of Chemistry , University of Liverpool , UK . Email: hardwick@liverpool.ac.uk; b Department of Physics , University of Liverpool , UK; c State Key Laboratory of Physical Chemistry and Solid Surfaces , University of Xiamen , China

## Abstract

Sodium–oxygen battery cathodes utilise the reversible redox species of oxygen in the presence of sodium ions. However, the oxygen reduction and evolution reaction mechanism is yet to be conclusively determined. In order to examine the part played by surface structure in sodium–oxygen electrochemistry for the development of catalytic materials and structures, a method of preparing clean, well-defined Pt electrode surfaces for adsorption studies in aprotic solvents is described. Using cyclic voltammetry (CV) and *in situ* electrochemical shell-isolated nanoparticle enhanced Raman spectroscopy (SHINERS), the various stages of oxygen reduction as a function of potential have been determined. It is found that on Pt{111} and Pt{110}-(1 × 1) terraces, a long lived surface sodium peroxide species is formed reversibly, whereas on Pt{100} and polycrystalline electrodes, this species is not detected.

## Abstract 2

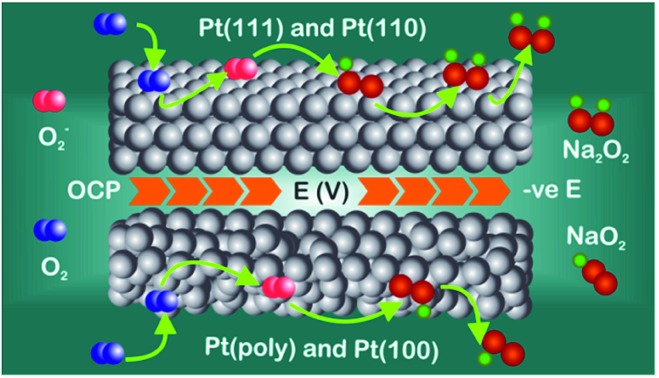
The oxygen reduction and evolution reaction in the presence of sodium ions in an organic solvent is studied on well-defined Pt electrode surfaces.

## Introduction

The development of sodium–oxygen (Na–O_2_) batteries has been of particular interest due to their large theoretical specific energies (1601 W h kg^–1^, Na_2_O_2_*vs.* 1106 W h kg^–1^, NaO_2_) (Table S1†).^1^ The reaction mechanisms of oxygen in aprotic solvents with alkali metal ions are, however, complex and involve the formation of highly nucleophilic and unstable reaction intermediates.^2^–^6^ Hence metal–O_2_ batteries in general such as Li–oxygen (Li–O_2_)^7^ and Na–oxygen (Na–O_2_)^8^ have yet to realise these notional specific energies over long term cycling.^1^ In this context, understanding of molecular processes at the electrode/electrolyte interface is vital.^8^

There have been a number of conflicting findings regarding the precise mechanism of oxygen reduction in the presence of sodium ions.^9^–^11^ A key parameter is reported to be the solvent used to dissolve the sodium-containing electrolyte.^12^,^13^ In addition, studies have usually been performed with polycrystalline electrode surfaces.^12^ However, in order to examine the part played by surface structure in sodium–oxygen electrochemistry, single crystal electrodes are preferred.

The ideal and controlled nature of using single crystal surfaces can provide information relating to adsorption properties^14^ such as particular adsorption sites^15^ and surface coverages.^14^ Although much work on the oxygen reduction reaction (ORR) in aqueous media using single crystal measurements has been reported,^16^–^19^ there have been relatively few non-aqueous single crystal studies, especially with regards to M–O_2_ batteries, and even these have usually been carried out on relatively unreactive gold surfaces.^20^,^21^

Studying the fundamental redox reaction mechanisms between oxygen and sodium cations at Pt{*hkl*} electrodes in aprotic solvents may provide further understanding of the contributions made by both bulk and surface redox processes in M–O_2_ batteries. Any electrocatalytic role played by platinum surface structure in facilitating oxygen reduction may also be examined in this way (influence of steps, kinks *etc.*) with the potential to study other well-defined surfaces of transition metals, surface alloys and bimetallics in a similar fashion.

## Experimental section

### Chemicals

NaClO_4_ (Aldrich) was dried under vacuum overnight at 90 °C and stored in an argon filled glovebox. DMSO was distilled to remove impurities then stored over molecular sieves (4 Å) for 1 week prior to use.

### SHINs synthesis

55 nm Au nanoparticles were synthesised using the standard sodium citrate reduction method.^22^ A uniform 2–3 nm silica shell was added to the Au nanoparticles using the method described previously.^23^,^24^

### Single crystal preparation

**Fig. 1:**
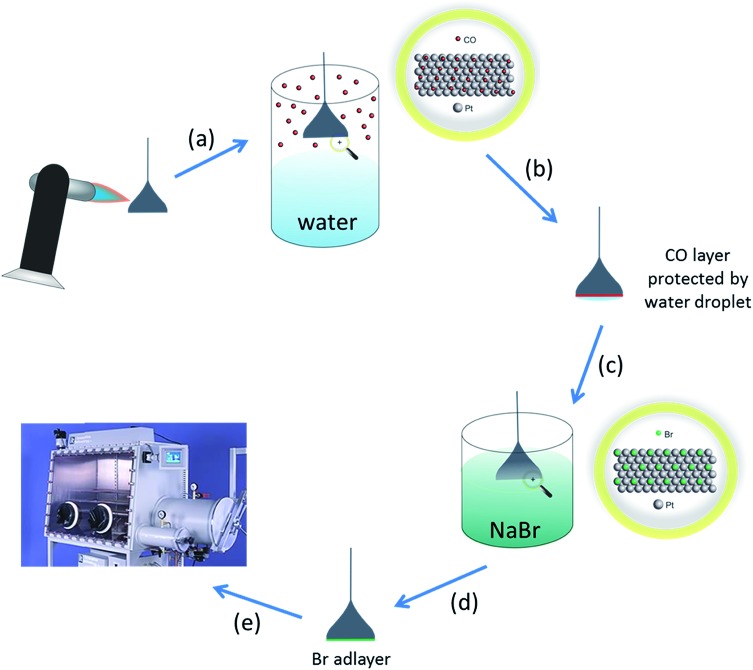
Schematic describing how single crystals were prepared for non-aqueous electrochemical studies. (a) Single crystal annealed to remove impurities from the electrode surface and re-order surface atoms. (b) Hot crystal cooled in CO atmosphere (adsorbed CO protects the surface from contamination and prevents oxidation)^26^ (N.B. for Pt{110}, CO also lifts the (1 × 2) missing row clean surface reconstruction leading to a well-ordered (1 × 1) phase after electrochemical stripping of the CO adlayer).^27^ (c) CO protected surface with a water droplet attached transferred to an aqueous NaBr solution. (d) Single crystal bead dipped in 1 mM NaBr solution forming a (4 × 4) bromine adlayer which protects the surface from contamination from the ambient.^28^ (e) After rinsing with pure water, the bromine adlayer-covered electrode is dried in glove-box chamber, ready to be used in electrochemical experiments. The bromine adlayer may be reductively desorbed in the electrochemical cell leaving behind the well-defined Pt{*hkl*} electrode surface.

Pt hemispherical bead single crystals were prepared using the Clavilier method.^25^ The crystals were cleaned by annealing in a butane flame followed by cooling in a CO/water atmosphere. The crystal was then protected from the laboratory ambient with a droplet of water before being dipped in a 1 mM solution of sodium bromide (NaBr). After rinsing with pure water to leave behind an irreversibly adsorbed layer of bromine atoms (which strongly inhibit any further chemisorption from the ambient), the crystal was dried in the glove box antechamber before use (technique demonstrated in Fig. 1). The amount of bromine adsorbed is approximately 7 × 10^14^ atoms per cm^2^ for a physical monolayer of bromine on Pt{111}, but since the crystal is 0.03 cm^2^ in area, the total number of bromine atoms to be desorbed is 0.03 × 7 × 10^14^ which is equal to 0.21 × 10^14^ atoms. Given the volume of the electrolyte in the electrochemical cell is approximately 30 cm^3^, a concentration of bromide ions can be estimated when desorbed of 1.162 × 10^–9^ M. This value is well below the mass transport limiting concentration for the adsorption of ions (which would be around 10^–6^ M). Hence, it is a reasonable assumption that no re-adsorption of bromide occurs onto the electrode surface after stripping during subsequent potential cycling. The Pt{*hkl*} CV measurements are extremely sensitive to both surface contamination and surface structure. The CVs of SHINs on the Pt facets demonstrates the SHINs have no effect on the electrochemical response (apart from a small deviation in current density due to site-blocking) (Fig. S1†).

### Electrochemical measurements

Electrochemical experiments were conducted using a potentiostat (Biologic) in an argon glovebox and an in-house glass cell. Dry O_2_ and Ar gas lines were used to bubble and purge electrolytes, with water contents of <20 ppm. All potentials were measured *vs.* Ag/Ag^+^ pseudo reference. The potentials were then calibrated with an internal Fc/Fc^+^ couple (0.68 V *vs.* SHE). The potentials of all data reported were then adjusted from the Fc/Fc^+^ to the Na/Na^+^ potential scale. Prior to experimentation, all glassware was cleaned in piranha solution (H_2_SO_4_ : H_2_O_2_ (5 : 1 v/v ratio)) and boiled three times in Milli-Q water. All glassware was dried under vacuum before use.

### Spectroscopic measurements

Raman experiments were performed using a Renishaw In-Via spectrometer with an inverted microscope and a 633 nm laser. A specifically designed glass cell with a sapphire window at the bottom was used. The cell was assembled in an argon filled glovebox, the electrolyte was purged with Ar to remove any gases, and then bubbled with O_2_ for 30 minutes to saturate the electrolyte, before being sealed and transferred to the Raman spectrometer.

### SHINERS experiment

2 μl of SHINs were drop cast onto Pt electrode surface. The electrode was left to dry in a N_2_ stream prior to use.

### Raman peak evaluation

Raman intensities were calculated by integrating the area under the desired peaks. The peaks were then normalised between 0 and 1 using a standard peak as the reference. The NaO_2_ peak (*ca.* 1155 cm^–1^) was chosen as it is present in all of the spectra, However, its intensity was normalised to unity within each individual spectra set (*i.e.* Pt{100}). This is because of varying Raman enhancements at different points on the surface. As the enhancement is dependent on the local distribution of the SHINs and may vary upon each deposition. Therefore, the level of enhancement may vary between experiments.

## Results and discussion

**Fig. 2:**
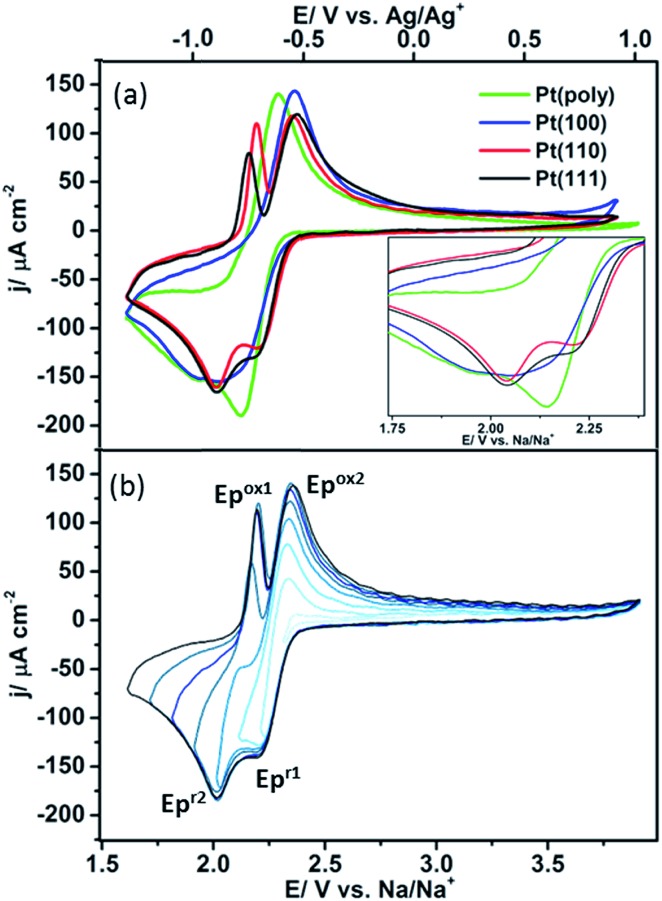
(a) Cyclic voltammetry of platinum single crystal facets Pt{111}, Pt{110}, Pt{100} and polycrystalline platinum in 0.1 M NaClO_4_, DMSO (O_2_ saturated). (Inset) Magnification of the reduction curve demonstrates that surface structure influences the overpotential for the onset of reduction. (b) Potential window opening on Pt{111} showing the association between the two surface redox states at 2.20 and 2.01 V. Sweep rate 50 mV s^–1^.

The oxygen reduction/oxidation voltammetric behaviour of the three basal platinum planes {111}, {110} and {100} together with polycrystalline platinum in 0.1 M NaClO_4_ dissolved in dimethyl sulfoxide (DMSO) is shown in Fig. 2a. Pt{111}, Pt{100} and Pt{110} facets were chosen since they are the simplest Pt surfaces containing only terraces. The voltammetric profiles on the facets remain stable after initial potential cycling to remove bromide adatoms (five cycles were sufficient to remove the bromide adlayer). After this point, the potential sweeps demonstrate the stability of the system towards solvent decomposition (*i.e.* upon cycling a further five cycles, a stable CV was produced indicating no subsequent breakdown into either molecularly adsorbed fragments or even possible surface reconstruction (Fig. S2†)). It is evident that the Pt{111} and Pt{110} electrodes display substantially different voltammetric behaviour compared to Pt{100} and polycrystalline platinum (Fig. 2a and Table S2†). Two peaks are observed in the reduction sweep on both {111} and {110}, a peak at ∽2.20 V followed by a second more intense feature at ∽2.01 V. In the positive-going oxidation sweep, sharp, distinct peaks at 2.16 V (Pt{111}) and 2.19 V (Pt{110}) are observed. Integrating the area under the 2.16 V peak for the Pt{111} electrode demonstrates a sub monolayer surface coverage of 0.41 (this was calculated assuming a 1e^–^ reduction (calculation after Fig. S3†)), using the data in Fig. S3† which confirmed an approximately 1e^–^ reduction was occurring (for a surface density of platinum atoms of 1.5 × 10^15^ cm^–2^). These narrow and intense features are completely absent when studying Pt{100} and polycrystalline platinum. In fact the Pt{100} surface displays a very broad reduction peak at ∽2.02 V, possibly consisting of three overlapping processes with the redox processes negative of 1.8 V largely absent from the Pt{110} and Pt{111} data (Fig. 2a). This suggests that the formation of the species at 2.0 V on Pt{110} and Pt{111} blocks the reduction processes occurring on the other two surfaces. This point will be discussed further in the next section. On the reverse sweep, a common, broader oxidation state at 2.37 V is observed for all Pt{*hkl*} and polycrystalline electrodes. The voltammetric peaks in the range 2.16–2.19 V on the positive-going scans which are a singular feature of sodium–oxygen voltammetry on platinum {111} and {110} imply that these surfaces are affording a unique surface reaction pathway. Later, it will be demonstrated spectroscopically that this is indeed the case.

The overpotential for the onset of the first oxygen reduction process varies between the different basal planes with Pt{110} (2.39 V) and Pt{111} (2.37 V) exhibiting the smallest overpotential and Pt{100} (2.34 V) the largest (Fig. 2a inset). In order to elucidate the possible association of redox peaks on both the positive- and negative-going sweeps for Pt{111}, a potential window opening experiment was performed with increasingly more negative potential limits (Fig. 2b). It is evident from Fig. 2b that two independent surface redox states may be identified, labelled Ep^ox1^/Ep^r2^ and Ep^ox2^/Ep^r1^ in the figure. This result diverges from results published by Peng *et al.*^11^ on a gold electrode, who demonstrated (Ep^r1^) was related to (Ep^ox1^), and (Ep^r2^) was associated with (Ep^ox2^).

**Fig. 3:**
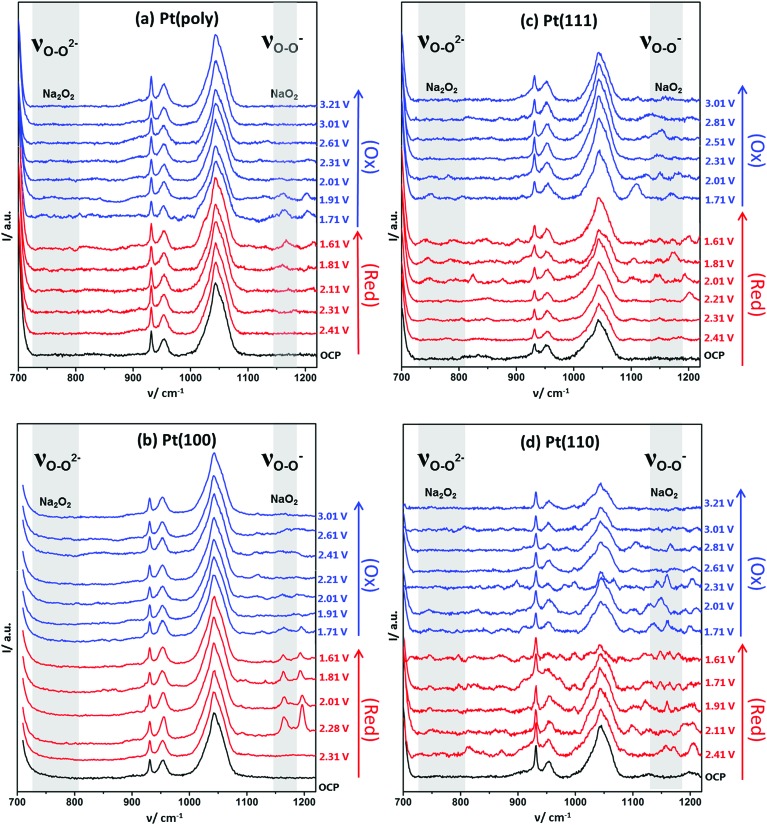
Full range spectra of EC-SHINERS of 0.1 M NaClO_4_ in DMSO on (a) polycrystalline Pt, (b) Pt{100}, (c) Pt{111} and (d) Pt{110}. All crystal surfaces prepared by flame-annealing and cooling in a CO/water gas stream followed by protection using an irreversibly adsorbed bromide monolayer before the addition of SHINERS nanoparticles. OCP was 2.8 V (key band assignment available in ESI Table S3†).

In order to elucidate the causes of the variations in electrochemical response between the different platinum surface arrangements, a spectroscopic study was conducted. *In situ* electrochemical shell-isolated nanoparticle enhanced Raman spectroscopy (EC-SHINERS)^24^,^29^ allowed the identification of molecular surface species formed as a function of potential and hence, the reaction mechanism between sodium ions and oxygen could be followed (Fig. 3 and Table 1). The SHINERS technique is invaluable for studying the reaction mechanisms as the SHINs particles are reliant on the strength of the electromagnetic field produced by the Au core of the particles. This Au core enhancement has a penetration depth of about 10 nm.^30^ Since the silica shell surrounding the nanoparticles is 2–3 nm thick, a maximum enhancement region of 7 nm around the circumference of the particles is deduced. The greatest enhancement is observed at the surface or between two nanoparticles.^31^ Therefore, (assuming surface selection rules are satisfied) the vibrational Raman signal of surface species are strongly enhanced, whilst also allowing the detection of solution species within a certain distance of the electrode surface.

**Table 1:** Corresponding Raman peak positions (cm^–1^) of 0.1 M NaClO_4_ in DMSO on different Pt single crystal facets from Fig. 3

Band assignment	Pt(111)	Pt(110)	Pt(100)	Pt(poly)	Literature
*ν*_O–O_(NaO_2_)_ads_	1154	1158	1162	1162	1156 (ref. 12 and 35)
*ν*_O–O_(Na_2_O_2_)	740	742	—	—	736 (ref. 35)
	798	798			791 (ref. 36)

The electrochemical response is independent of the sodium salt used, providing the anion is stable in the presence of the superoxide species and does not degrade on the platinum surface (Fig. S4 and S5†). Furthermore, similar electrochemical behaviour has also been observed in a sulfolane/DMSO electrolyte blend (Fig. S6†). However, finding alternative solvents that are compatible with the Pt surface has proved difficult due to their surface instability or blocking effect, *i.e.* MeCN adsorbs strongly onto the platinum surface inhibiting the reduction of surface oxygen species, resulting in a diminution in both the voltammetric differences between the various Pt{*hkl*} electrodes and the absolute charges associated with Na–O_2_ surface electrochemistry (Fig. S7†).^32^ Moreover, association of the unique CV responses of Pt{*hkl*} with local surface structure may be further demonstrated *via* the blocking of surface sites by irreversibly adsorbed bromide anions from the electrolyte phase (Fig. S8†) whereby 1 mM NaBr was added into a 0.1 M NaClO_4_/DMSO electrolyte. The addition of bromide (Br^–^) causes the anion to be irreversibly adsorbed onto the Pt{110} electrode surface (Fig. S8†). As the Br^–^ coverage increases with cycling (exposure time), it is observed that ORR charge density for the Na–O_2_ system diminishes. However, at least in the initial stages of bromide uptake, the structure sensitive CV profile reported in Fig. 2 is retained (unlike in the case of acetonitrile decomposition). Almost complete quenching of voltammetric oxidation peaks at highest bromide coverages attests to the requirement of free Pt surface sites in the mechanism of superoxide and peroxide formation.

In Fig. 3 the changes in the *in situ* SHINERS corresponding to the potential cycle recorded under identical conditions to Fig. 2a are shown for polycrystalline Pt. At the open circuit potential (OCP), bands for the electrolyte are observed, most notable bands for DMSO at 955 and 1044 cm^–1^ and for ClO_4_^–^ at 930 cm^–1^. Vibrational bands at 1115 cm^–1^ and 1162 cm^–1^ may be ascribed to the formation of adsorbed free superoxide (non-coordinated or weakly coordinated O_2_^–^ species) and sodium superoxide respectively.^11^,^23^,^33^,^34^

On the Pt {100} surface, the sodium superoxide species is formed initially at 2.28 V. Upon reversing the direction of the potential sweep, it is found that the desorption of sodium superoxide occurs. Hence, in Fig. 2, one may positively identify the species formed initially at 2.28 V as corresponding to the formation of sodium superoxide. The vibrational modes of Na_2_O_2_ and NaO_2_ are well documented in the literature,^12^,^37^ yet no other surface species are observed, such as peroxide-type adsorbates, which would typically give rise to Raman peaks around 736 and 792 cm^–1^.^12^,^36^

When polycrystalline Pt is considered, it appears to give a very similar spectroscopic response to the {100} platinum electrode surface (Fig. 3). For example, distinct Raman features ascribable to superoxide and sodium superoxide at 1115 cm^–1^ and 1162 cm^–1^ respectively are clearly observed. Again, it is evident that initial formation of a sodium superoxide species occurs. The sodium superoxide peak is shifted to slightly higher wavenumber values than previously reported for solid NaO_2_ (1156 cm^–1^) on a gas diffusion layer cathode.^38^ Nonetheless, in relation to the voltammetry reported in Fig. 2a, no spectroscopic signatures of other oxygen-containing species are reported in Fig. 3. The voltammetric peak at the most positive potential on Pt{100} is ascribed to sodium superoxide formation.

**Fig. 4:**
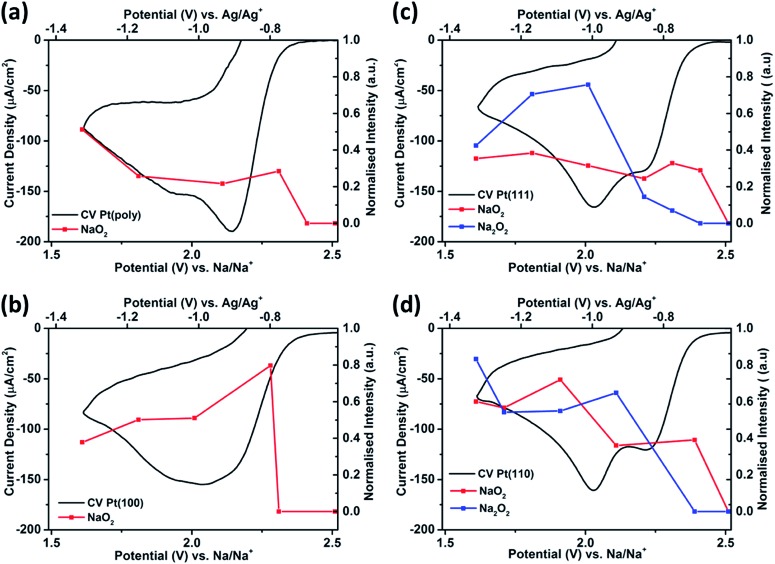
CVs of the reduction region of ORR and corresponding potential dependent Raman intensities* for NaO_2_ (1160 cm^–1^) and Na_2_O_2_ (740 cm^–1^) from SHINERS data in 0.1 M NaClO_4_ in DMSO (saturated with O_2_) on (a) Pt(poly), (b) Pt{100}, (c) Pt{111} and (d) Pt{110} (*Raman intensities, integrated area under the desired peaks from Fig. 3 and then all peaks normalised between 0 and 1 using the NaO_2_ peak in Pt{100} at 1.81 V as the reference).

Fig. S9† illustrates clearly the changes observed in the SHINERS data (Fig. 3) arising from Pt{111} under the same conditions as for Pt{100} and polycrystalline platinum. In this case, the spectra recorded demonstrates the growth of peroxide on the {111} surface. For example, at 740 cm^–1^, the appearance of a sodium peroxide peak is clearly observed as the potential is swept more negatively from 2.01 V. In fact, a strongly correlated peak formation at 798 cm^–1^ may also be identified as being due to the sodium peroxide stretch within the solid crystal^36^ exhibiting a correspondingly reduced intensity compared to the 740 cm^–1^ peak.^12^ Hence, it appears that a “molecular” absorbed form of a sodium peroxide species (Na_2_O_2ads_ and/or NaO_2ads_^–^) forms initially (single Raman stretch at 740 cm^–1^) followed by formation of bulk sodium peroxide (peaks at both 740 cm^–1^ and 798 cm^–1^) as potential becomes more negative. A similar trend is observed on the Pt{110} surface (Fig. 3a) with an initial peak at 742 cm^–1^ from 2.11 V followed by an additional stretch at 798 cm^–1^ from 1.91 V, both ascribed to sodium peroxide. In addition to the sodium peroxide phase, peaks ascribable to superoxide (1107 cm^–1^ Pt{111} and 1105 cm^–1^ Pt{110}) and sodium superoxide (1154 cm^–1^ Pt{111} and 1158 cm^–1^ Pt{110}) are formed (Fig. 3). However, it is clear that the spectra in Fig. 3 are more complex and guided by our previous work on Li–O_2_ and Na–O_2_ in the same electrolyte (including calculations of vibrational frequencies),^39^ we tentatively assign the peaks as *ν*_O–O_ of NaO_2_H (824 cm^–1^), H_2_O_2_ (874 cm^–1^) and HO_2_ (1190 cm^–1^) formed as a consequence of trace water (15–20 ppm) being present in the electrolyte. However, in contrast to polycrystalline platinum and Pt{100} it is obvious that for Pt{111} and Pt{110} a reactive surface pathway involving the formation of surface peroxy-species is available (Fig. 4 and S10†), which in turn may generate extra species associated with water impurities. The potential ranges in which all of these new species (in addition to superoxide and sodium superoxide) form corresponds to the reversible surface redox peak seen exclusively with Pt{111} and Pt{110} in Fig. 2a.

**Fig. 5:**
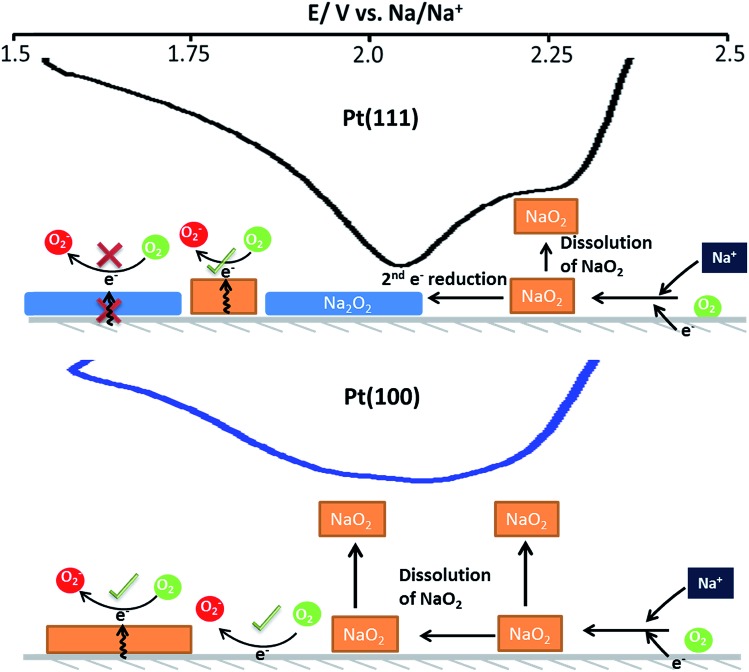
Schematic of the reaction pathways on the Pt(111) and Pt(100) crystal facets.

A suggested mechanism for all reactions is schematically shown in the Fig. 5 whereby three surface oxygen reduction processes may occur. We do not discount the possibility of further reduction/chemical reactions occurring in the solution phase but concentrate on the surface processes reported for the first time in the present study:

(1) Reduction of oxygen in the presence of sodium ions to form surface sodium superoxide at free Pt sites.

(2) Conversion of surface sodium superoxide to sodium peroxide on Pt{111} and Pt{110}.

(3) Conversion of oxygen to superoxide at sites blocked by adsorbed sodium superoxide.

**Fig. 6:**
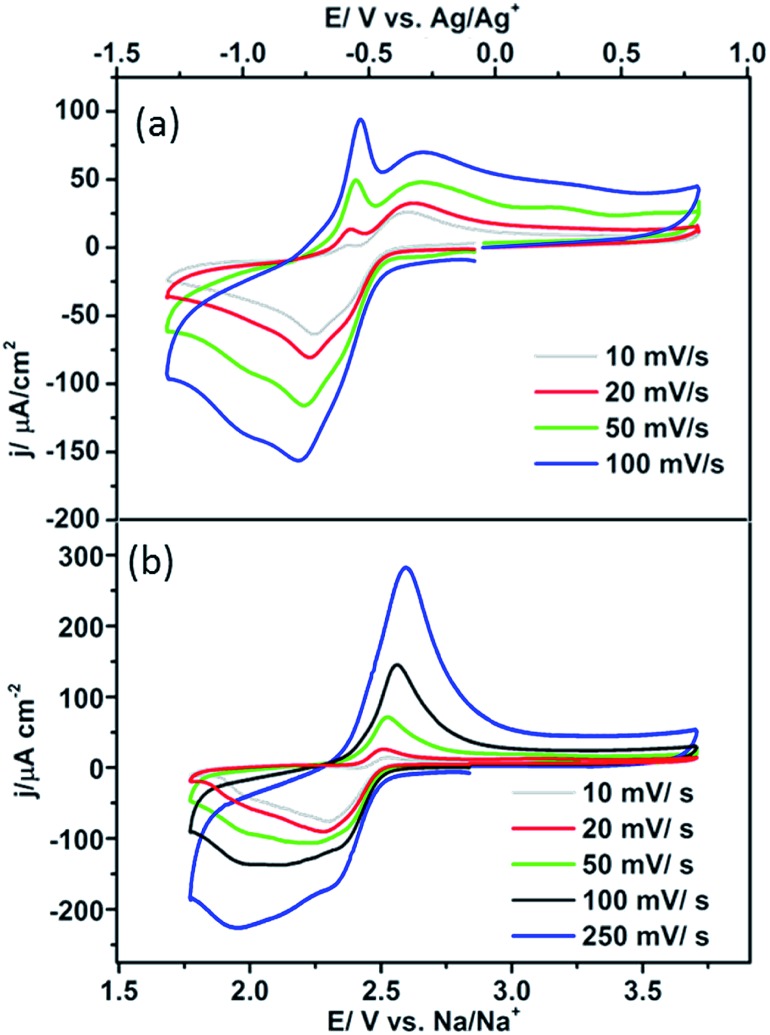
(a) CVs of 1 M NaClO_4_ in DMSO (saturated with O_2_) on Pt{111} at different sweep rates, (b) CVs of 0.1 M NaClO_4_ in DMSO (saturated with O_2_) on Pt{100}, at different sweep rates.

In this interpretation, the broad reduction peak observed on polycrystalline Pt and Pt{100} consists of a number of overlapping processes (Fig. S11†), which are surface sodium superoxide and sodium peroxide formation followed at more negative potentials by reduction of oxygen to superoxide at sodium superoxide covered platinum sites. The blocking of this later process on Pt{111} and Pt{110} referred to previously may then be interpreted as reflecting a difference in electrical conductivity between NaO_2_ and Na_2_O_2_ with the peroxide phase reported to be much more insulating than the superoxide.^40^ In order to help further elucidate both the presence of Na_2_O_2_ and the stability of the Na_2_O_2_ species on the Pt{*hkl*} electrode surface, a sweep rate study was undertaken (Fig. 6a). Note that the increase in sodium perchlorate concentration from 0.1 M to 1 M for Pt{111} allowed for a decrease in the amount of surface sodium peroxide formed (attenuation in sharp sodium peroxide peak intensity relative to that in Fig. 2) and a concomitant increase in superoxide formation at more negative potentials due to decreased blocking by “insulating” surface sodium peroxide (see later for peak deconvolution analysis). Previous analysis for Pt{110} and Pt{111} demonstrated that the sharp oxidation/reduction couple in Fig. 2 can be attributed to sodium peroxide species. For both of these surfaces, the sweep rate dependence of peak intensity is linear *versus* sweep rate for the ‘sharp’ oxidation peak indicating a surface based reaction, namely the 1e^–^ oxidation of Na_2_O_2_. The second oxidation peak gives rise to a square root dependence on sweep rate showing this peak to be a solution based diffusional process (Fig. S3†). A similar sweep rate study was undertaken on the Pt{100} facet (Fig. 6b). Fig. 6b demonstrates that with increasing sweep rate, a splitting of the peak in the reduction process is observed. The second reduction process becomes much stronger relative to the first with an increase in sweep rate. In addition, the balance of overall cathodic and anodic charge rapidly changes as sweep rate is reduced such that the oxidation charge process is approximately 25 times smaller than the reductive charge at 10 mV s^–1^. This would suggest that at such slow sweep rates, diffusion of superoxide/sodium superoxide species away from the electrode has occurred before it can be oxidised on the positive-going sweep. Hence, one would predict that as a function of time, the SHINERS signal from adsorbed NaO_2_ on Pt{100} might decrease. This is indeed confirmed in Fig. 6b showing a net decrease in NaO_2_ intensity as potential is decreased (also time increases). Hence, we assert that NaO_2_ is dissolved from the electrode surface over time. However, the presence of a long-lived sodium peroxide phase on the {100} facet is not detected. The potentials of the first two reduction peaks coincide with sodium superoxide and sodium peroxide formation as deduced using Pt{110} and Pt{111} (Fig. S11†). This suggests that sodium superoxide may also be formed on Pt{100} but that it rapidly diffuses away from the surface once formed and has negligible lifetime on the Pt{100} surface. Deconvolution of the Pt{100} reduction peak at a sweep rate of 100 mV s^–1^ in Fig. 6b, into three peaks, the first two of these centred at the potentials of sodium superoxide and sodium peroxide on Pt{111} and Pt{110} and the residual peak at most negative potentials (assigned to pure superoxide) allows for the relative amounts of each reduction product to be assessed (Fig. S11 and Table S4†). It is evident from these deconvolution measurements that the proportion of sodium peroxide/sodium superoxide is small for Pt{100}.

Hence, a weak interaction of the surface peroxide species with the Pt{100} surface is suggested. The EC-SHINERS data for Pt{100} and polycrystalline platinum do not indicate the presence of Na_2_O_2_ at any potential and this implies that the 2^nd^ electron reduction of the NaO_2_ species is either not occurring on these surfaces or more likely, as confirmed by potential sweep rate measurements, that once formed, Na_2_O_2_ rapidly dissolves and leaves the Pt{100} surface.

Evidence for the weaker interaction of oxygen with Pt{100} compared to Pt{111} (and Pt{110}) has also been reported from density functional theory (DFT) and surface science studies.^41^ DFT studies have suggested that Pt{111} has the strongest adsorption energy for oxygen of the three basal planes, with oxygen preferentially adsorbing at bridge sites *i.e.* O–O bond lies between platinum atoms, and each oxygen atom covalently bonded atop platinum atoms.^42^,^43^ Studies in aqueous media (0.1 M KOH or 0.1 M HClO_4_) have also highlighted Pt{100} as the least active surface.^44^ These values all represent O_2_ not O_2_^–^, but a similar trend has been assumed in relation to surface activity for the superoxide species.^45^ This interpretation could afford an intrinsic reason for the voltammetric differences observed in the present study.

The ability to steer the progression of ORR towards superoxide or peroxide will have very important consequences for future battery design with obvious implications not only for charge storage but also for the stability of all components constituting the matrix of materials governing the efficacies of Na–O_2_ batteries.

Both NaO_2_ and Na_2_O_2_ are viable reduction products, albeit thermodynamically Na_2_O_2_ is the more stable discharge product with a free energy change (Δ*G*) of –449.7 kJ mol^–1^ compared to –218.75 kJ mol^–1^ for the superoxide species.^46^ However, it has been commonly suggested that NaO_2_ is the predominant reduction species as it is kinetically more favourable.^2^,^38^,^47^,^48^ Studies have also demonstrated that Na_2_O_2_ is a possible product, but the species is usually formed in a hydrated state.^9^,^47^,^49^,^50^

The initial reduction peak at ∽2.20 V is assigned to the 1e^–^ reduction of oxygen in the presence of sodium to form a sodium superoxide species on the electrode surface (eqn (1)). Eqn (2) (O_2_ → O_2_^–^) also occurs at greater over-potentials as dissolution of NaO_2_ occurs from the surface or reduction of oxygen at surface sites without Na^+^, when the Na^+^ cation concentration is limited by diffusion.

1 O_2(ads)_ + e^–^ + Na^+^ → NaO_2(ads)_

2 O_2_ + e^–^ → O_2(ads)_˙^–^

3 NaO_2(ads)_ + e^–^ + Na^+^ → Na_2_O_2(ads)_ (2^nd^ e^–^ transfer)

As mentioned earlier there is a disparity reported in the literature for the identification of the discharge product. It has been well documented that the electrolyte is an important factor in determining whether NaO_2_ or Na_2_O_2_ is formed. Low donor number solvents have been shown to promote Na_2_O_2_ as rapid disproportionation can occur.^37^ Alternatively it is shown that high donor number solvents, such as DMSO (29.8) promote the formation of NaO_2_.^12^,^37^

The electrolyte media has an important part to play in the reaction mechanism, however it is hypothesised that depending on the electrode surface structure/composition that either NaO_2_, Na_2_O_2_ or a combination of both can be formed as the discharge product.^9^ It has been proposed that the direct reduction of surface adsorbed O_2_ to NaO_2_ was occurring on a gold surface.^11^ Alternatively, NaO_2(ads)_ undergoes a second electron reduction to form initially Na_2_O_2_ (eqn (3)) on platinum, with the co-adsorption of superoxide on the electrode surface with sodium allowing formation of peroxide (predominately on the {111} and {110} planes). Furthermore, the dissolution of NaO_2_ (ads → sol) from the electrode surface in the presence of highly solvating DMSO solvent, on the less active surfaces (poly and {100}) precludes further reduction of NaO_2_, which ties in with results reported in the literature,^37^ as this process is confined to the electrode surface.

## Conclusion

A method for the preparation of {110}, {100} and {111} platinum single crystal electrodes for use in non-aqueous studies has been demonstrated. Electrochemical and spectro-electrochemical studies showed that the ORR on platinum is surface specific. On the {110} and {111} platinum basal planes the reduction of NaO_2_ to Na_2_O_2_ is promoted. This phenomenon is not observed on the Pt{100} or polycrystalline surfaces, likely related to the limited surface interaction with adsorbed oxygen compared to Pt{111} and Pt{110}. The discovery that Pt surfaces can control the reaction pathway between superoxide and peroxide species opens up the development of practical electro-catalysts capable of controlling the desired reaction pathway towards Na_2_O_2_ to deliver Na–O_2_ batteries with higher theoretical specific energies.

## Associated content

Corresponding peak positions and assignments for Fig. 2 and 3. CV's of DMSO with different supporting salts and electrolytes and electrolyte blends. CV of the blocking effect of excess Br ions. CV's with Raman intensities for Fig. 3.

## Abbreviations

SHINERS: shell-isolated nanoparticle for enhanced Raman spectroscopy

## Conflicts of interest

The authors declare no competing financial interest.

## Supplementary Material

Supplementary information
