# Supplementary material for: Oxygen reactions on Pt{hkl} in a non-aqueous Na+ electrolyte: site selective stabilisation of a sodium peroxy species
Source: Chem Sci. 2019 Jan 17;10(10):2956–64. doi: 10.1039/c8sc05489d (PMC6427968; doi:10.1039/c8sc05489d)
Supplement: Supplementary file 1 [file SC-010-C8SC05489D-s001.pdf]

Electronic Supplementary Material (ESI) for Chemical Science.
This journal is © The Royal Society of Chemistry 2019

Supporting Information

Oxygen reactions on Pt{hkl} in a non-aqueous Na^+ electrolyte: Site selective stabilisation of a sodium peroxy species

Thomas A. Galloway[a], Jin-Chao Dong[c], Jian-Feng Li[c], Gary Attard [b]

and Laurence J. Hardwick* [a]

*hardwick@liverpool.ac.uk

[a] Stephenson Institute for Renewable Energy, Department of Chemistry, University of Liverpool, Liverpool, L69 7ZD (UK)

[b] Department of Physics, University of Liverpool, Liverpool, L69 7ZD (UK)

[c] State Key Laboratory of Physical Chemistry and Solid Surfaces, University of Xiamen (China)

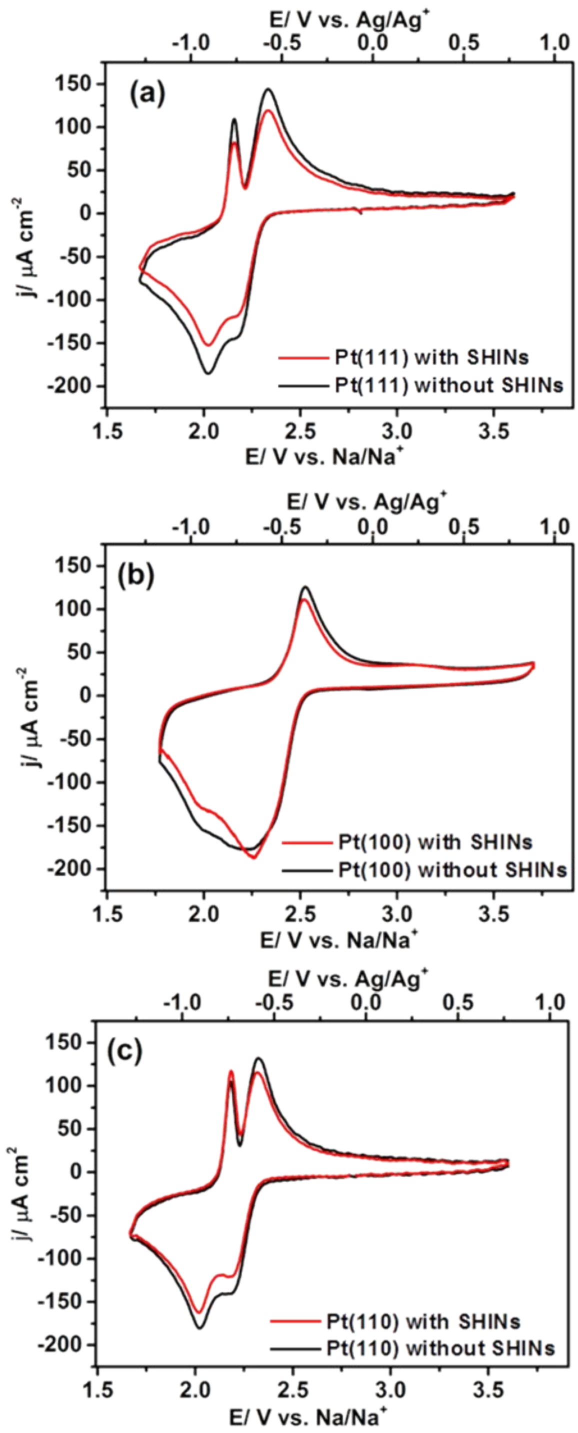

Figure S1. CVs of 0.1 M $NaClO_4$ in DMSO on different Pt single crystal facets with SHINS (red) and without SHINs (black) on (a) Pt {111}, (b) Pt {100} (c) Pt {110}. Sweep rate 50 mV/s.

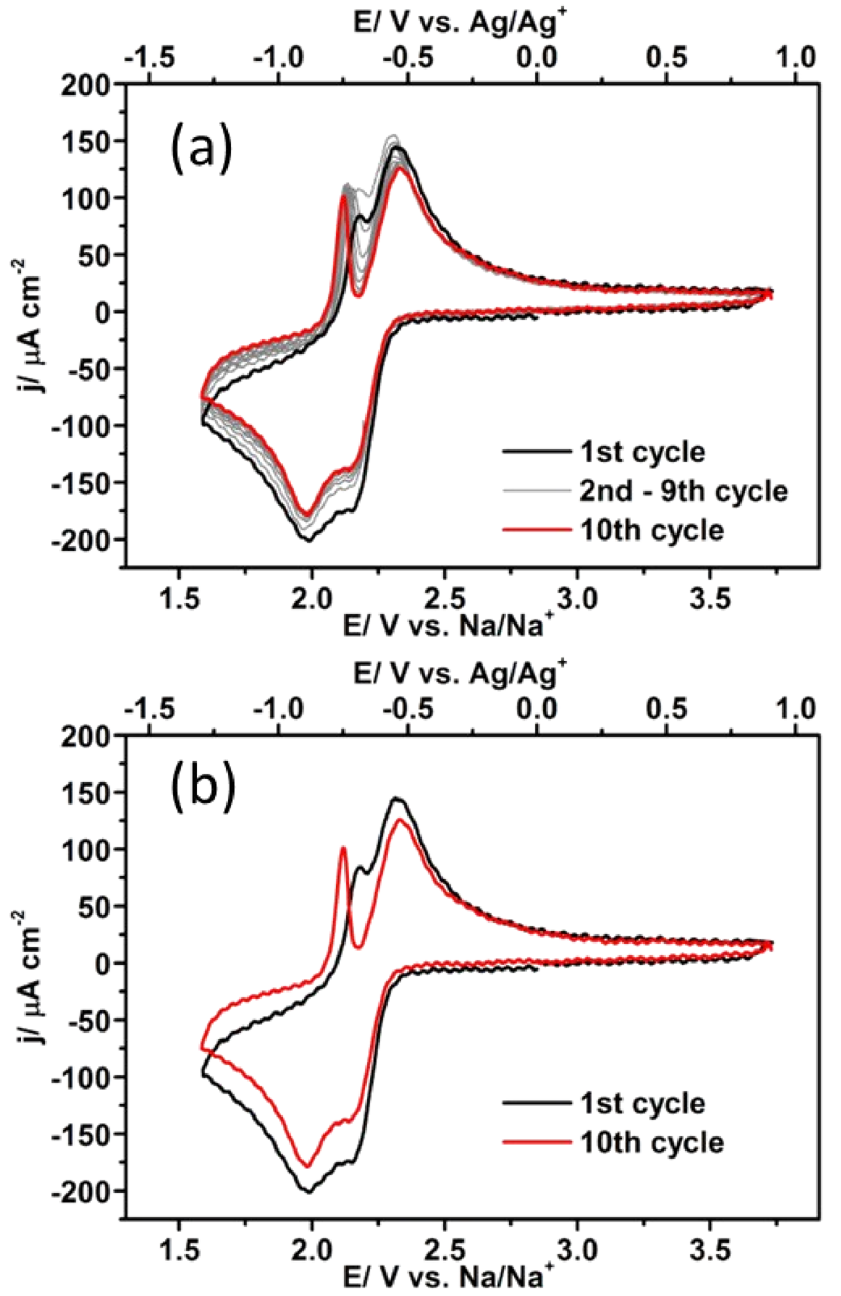

Figure S2 **(a)** CVs of 0.1 M $NaClO_4$ in DMSO (saturated with O_2) on Pt(111) 10 cycles, **(b)** CV showing 1st and 10th cycle. Sweep rate 50 mV/s.

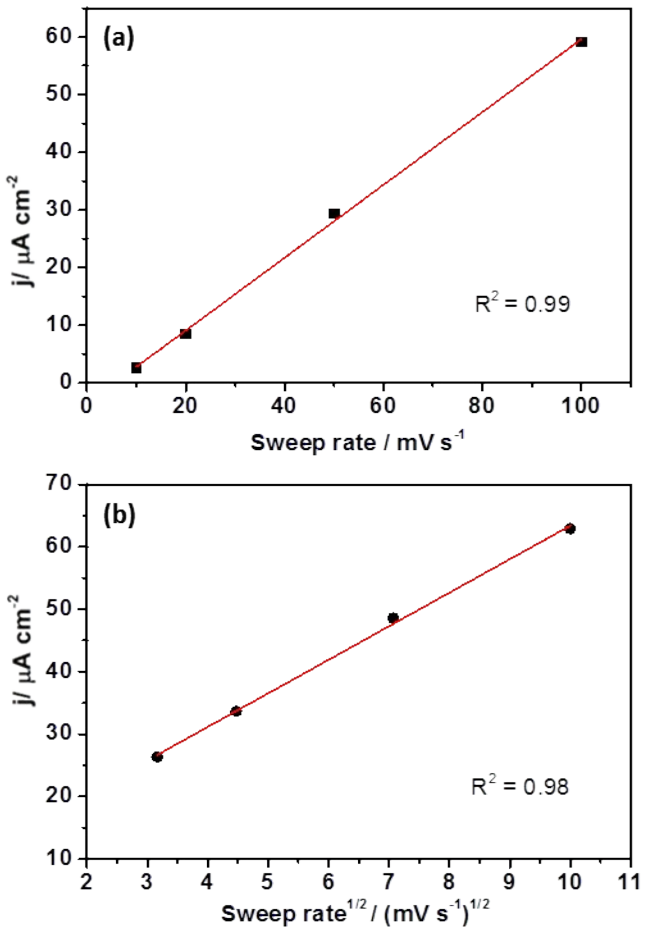

Figure S3. Plots of CV peak intensities as shown in Figure 5a for (a) 1st oxidation peak vs. sweep rate, (b) 2nd oxidation peak vs. sweep rate$^{1/2}$. Data taken from CVs of 0.1 M $NaClO_4$ on Pt{111} (Figure 5a).

Using equation 14.3.12 from Bard and Faulkner[1] (Eq. S1), the relationship between peak current and sweep rate for an adsorbed species.

Eq. S1 $$i_p = \frac{n^2F^2\nu A\Gamma_o^*}{4RT}$$

Eq. S2 $$Q^* = nFA\Gamma_O^* = \text{total adsorption charge}$$

Eq. S3 $$i_p = \frac{nFvQ^*}{4RT}$$

Gradient (m) from plot i_p vs. ν is

Eq. S4 $$m = \frac{nFQ^*}{4RT}$$

Therefore n can be calculated using the gradient from Figure S3a = 6.3 x 10^{-2} $CV^{-1}cm^{-2}$, Q^* (C cm^{-2}) (calculated from Figure 6a, Q^* = 76 μC cm^{-2}), F is Faraday's constant (96485 C mol^{-1}), R is the molar gas constant (8.314 J mol^{-1} K^{-1}) and T the temperature (293 K).

n = 0.851 , this correlates to approximately a 1 e^- transfer process occuring at the surface for the 1st oxidation peak, in agreement with the oxidation of Na_2O_2 to NaO_2.

.

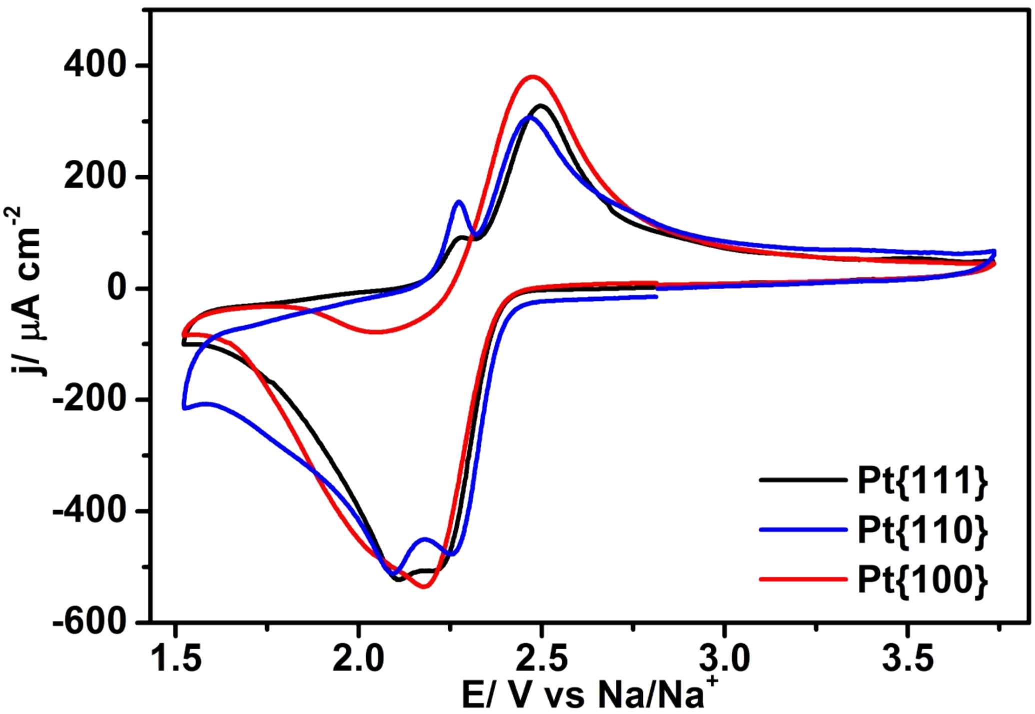

Fi

gure S4. CVs of 0.1 M NaTFSI in DMSO on different Pt single crystal facets. Sweep rate 50 mV/s.

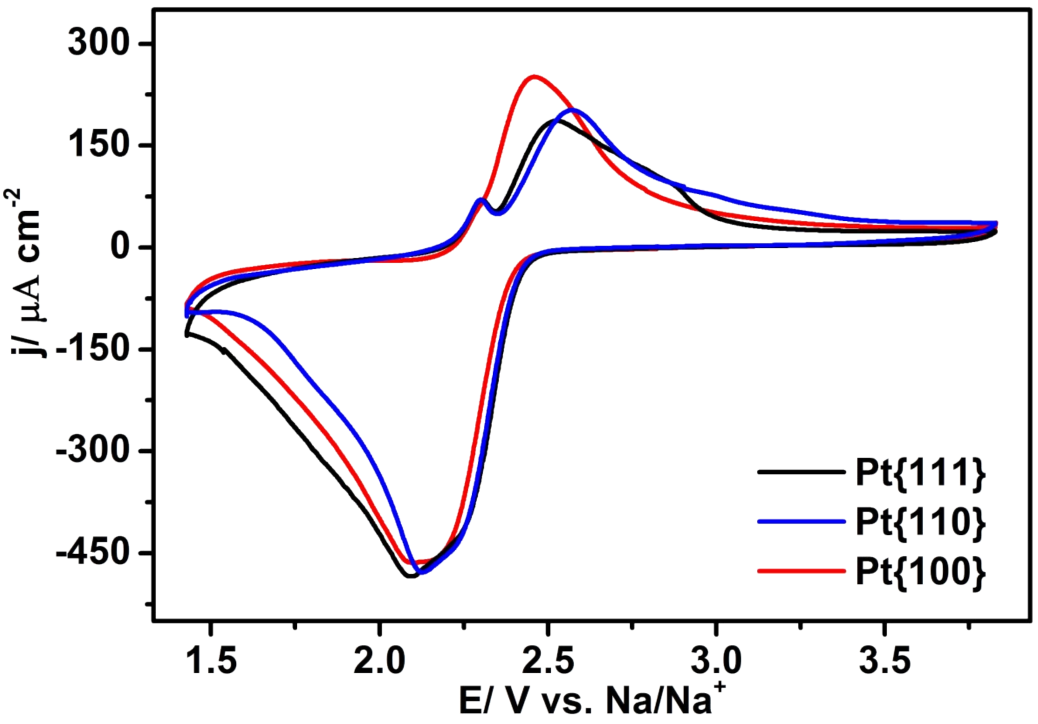

Figure S5. CVs of 0.1 M NaOTF in DMSO on different Pt single crystal facets. Sweep rate 50 mV/s.

Figures S2 and S4-S5 demonstrate that the ORR occuring on the platinum single crystal facets are independent of the Na salt used, providing the anion is stable in the presence of superoxide species and does not degrade on the platinum surface. The use of sodium OTf and sodium TFSI as alternative anions produced a similar electrochemical trend to that of perchlorate. A sharp initial oxidation peak was observed on the Pt{111} and Pt{110} surfaces. The Pt{100} surface only displayed a single oxidation peak in agreement with the perchlorate data. Figure S6 demonstrates that the same reaction mechanism also occurs in an alternative solvent. However the solvents suitable for use on Pt are limited (Figure S7).

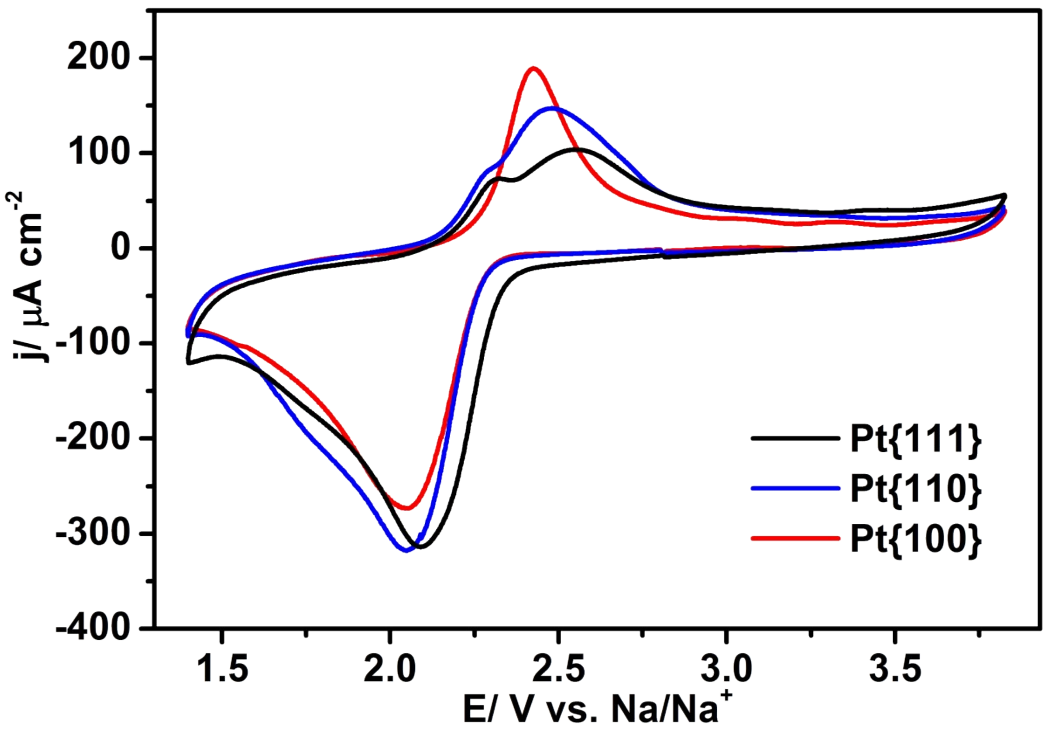

Figure S6. CVs of 0.1 M NaOTF in DMSO/ sulpholane electrolyte blend (2:1) on different Pt single crystal facets. Sweep rate 50 mV/s.

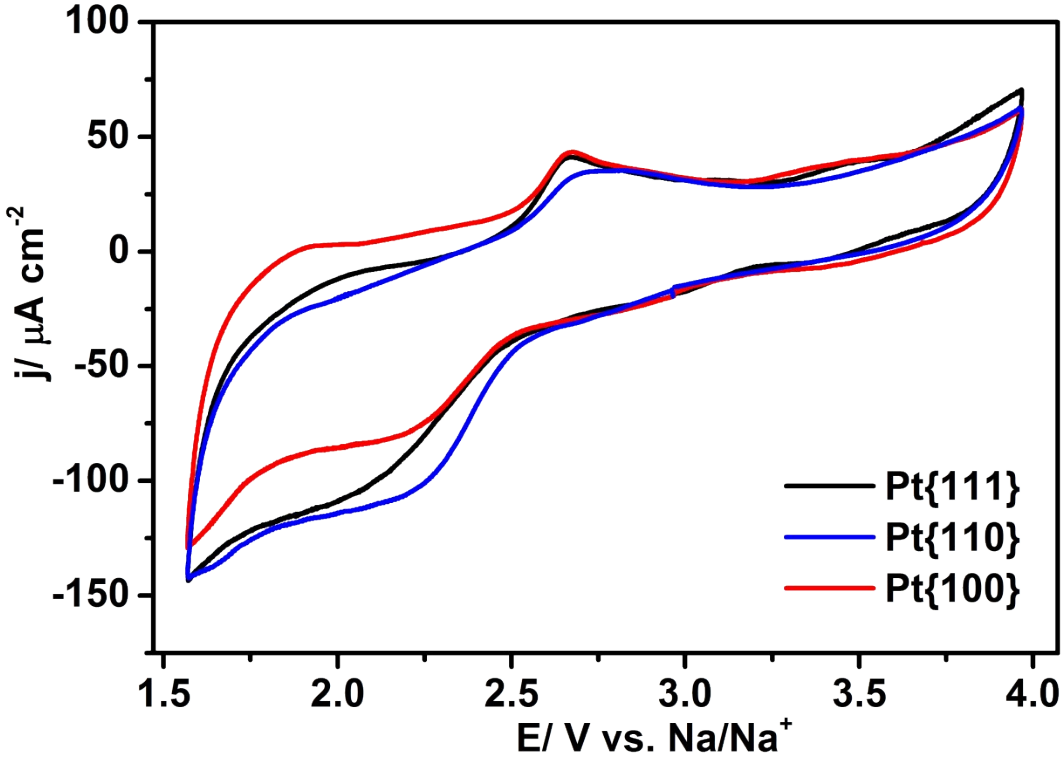

Figure S7. CVs of 0.1 M $NaClO_4$ in MeCN on different Pt single crystal facets. Sweep rate 50 mV/s.

Figure S7 illustrates the importance of the stability of the interface on the oxygen reduction reaction (ORR). Acetonitrile is well documented to form a blocking layer on the electrode surface,[2, 3] passivating the ORR at the electrode surface which is dependent on free surface Pt sites.

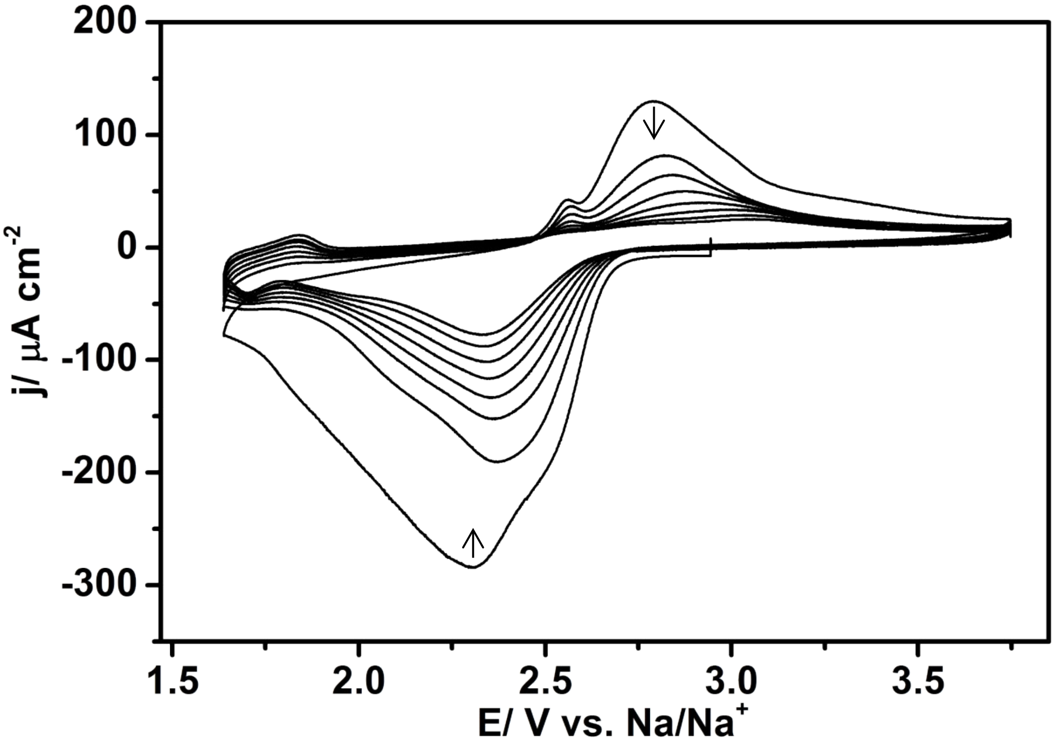

F

igure S8. CV of 1 mM NaBr in 0.1 M $NaClO_4$/ DMSO on Pt{110}. Sweep rate 50 mV/s.

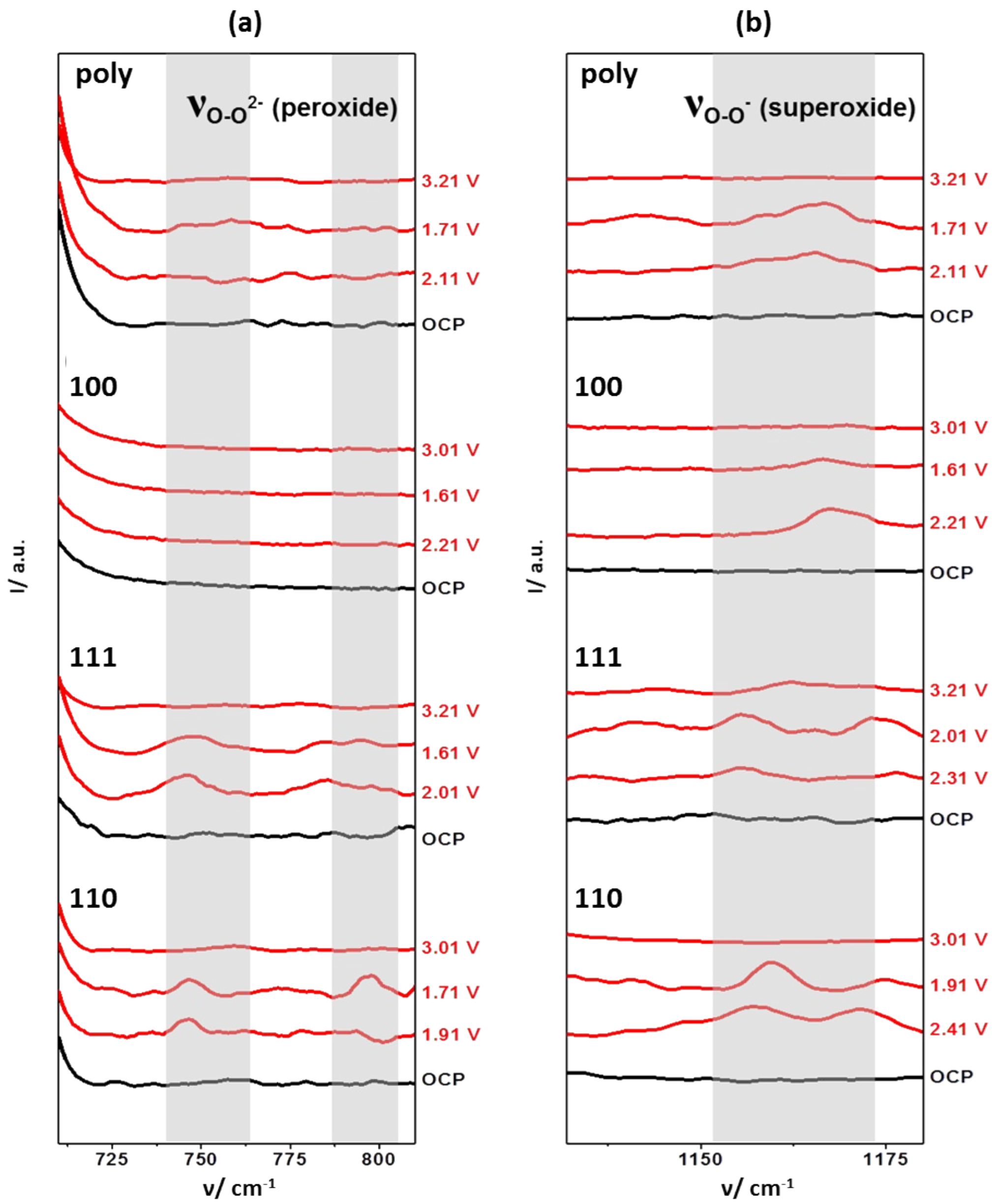

Figure S9. EC-SHINERS of 0.1 M $NaClO_4$ in DMSO on Pt poly, Pt{100},Pt{111} and Pt{110} (a) peroxide vibrational modes region (b) superoxide vibrational modes region . All surfaces prepared by flame-annealing and cooling in a CO/water gas stream, then protected using an irreversibly adsorbed Br^- monolayer before addition of SHINs nanoparticles. OCP was 2.8 V. (Key band assignment available in supplementary information **Table S3**).

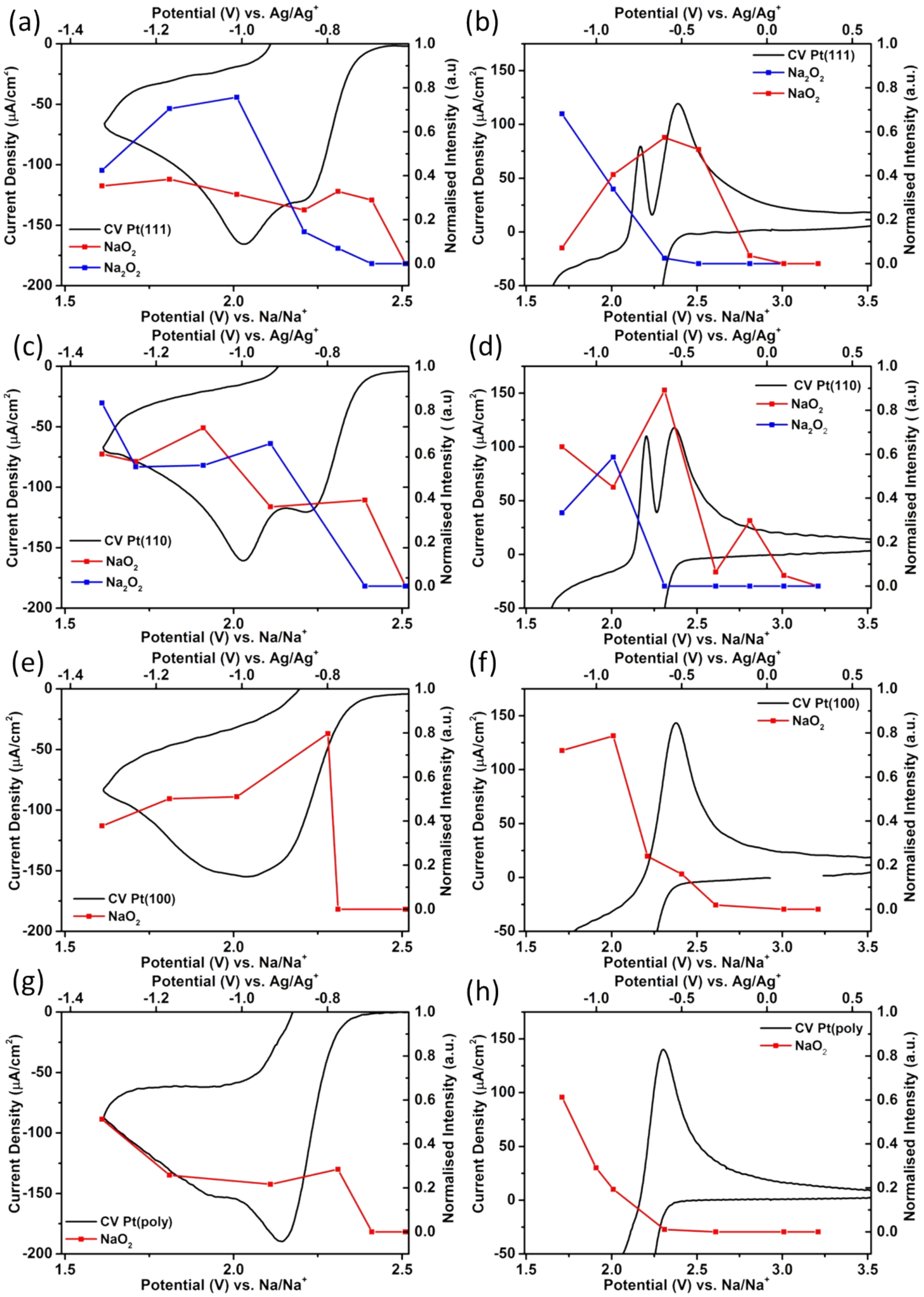

Figure S10. CVs with Raman intensities of νO-O of NaO_2 (1158cm^{-1}) and Na_2O_2 (740 cm^{-1}) in 0.1 M $NaClO_4$/DMSO on (a,b) Pt{111}, (c, d) {110}, (e,f) {100} and (g,h) poly. (a,c,e,g reduction Raman intensities and b,d,f,h oxidation Raman intensities)

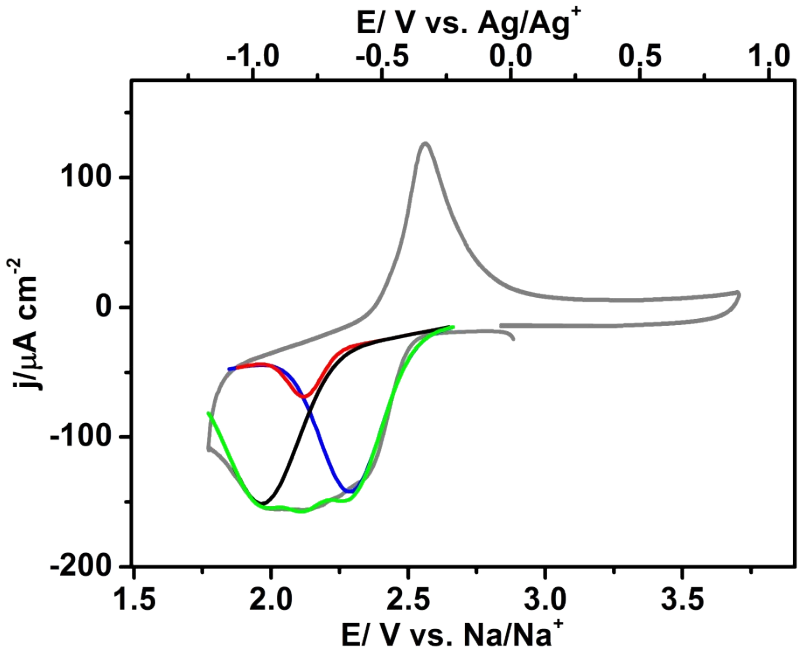

Figure S11. Peak fitting of 0.1 M $NaClO_4$ in DMSO on Pt{100}. Sweep rate 100 mV/s. The blue curve corresponds to sodium superoxide formation, the red curve to sodium peroxide and the black curve to superoxide formed from oxygen reduction on sodium superoxide covered platinum sites.

Table S1. Theoretical Specific Energies for $Na\text{-}O_2$

**Battery (theoretical values)**	**ΔG/ kJ mol^{-1}**	**Potential/ V**	**Specific Energy/ Wh kg^{-1}**
$Na\text{-}O_2$ (non-aq):			
$Na + O_2 = NaO_2$	- 218.4	2.27	1106
$2Na + O_2 = Na_2O_2$	- 449.7	2.33	1601

The theoretical specific energy for the reaction to form the peroxide (Na_2O_2) involving a 2 electron reduction is greater (1601 Wh kg^{-1}) than to the formation of the superoxide (NaO_2) via a 1 electron reduction (1106 Wh kg^{-1}). Values in table calculated directly from open source thermodynamic data.

Table S2. Corresponding peak positions of 0.1 M $NaClO_4$ in DMSO on different Pt single crystal facets from Figure 2a and CV data from Figures S1 and S4-S7. Potentials vs. Na/Na^+.

		Redox peaks observed in CVs			
**Electrode/ Electrolyte**		**Ep^{r1}**	**Ep^{r2}**	**Ep^{ox1}**	**Ep^{ox2}**
**0.1 M $NaClO_4$/ DMSO**	**Pt{111}**	2.20 V	2.01 V	2.16 V	2.37 V
	**Pt{110}**	2.21 V	2.02 V	2.19 V	2.39 V
	**Pt{100}**	2.02 V (broad)		-	2.34 V
	**Pt{poly}**	2.12 V	1.95 V	-	2.29 V
**0.1 M NaTFSI/ DMSO**	**Pt{111}**	2.22 V	2.10 V	2.27 V	2.49 V
	**Pt{110}**	2.25	2.08 V	2.28 V	2.46 V
	**Pt{100}**	2.17 V	2.02 V	-	2.49 V
**0.1 M NaOTF/ DMSO**	**Pt{111}**	2.23 V	2.08 V	2.28 V	2.50 V
	**Pt{110}**	2.24 V	2.10 V	2.28 V	2.53 V
	**Pt{100}**	2.19 V	2.08 V	-	2.43 V
**0.1 M NaOTF/ 2D:1S***	**Pt{111}**	2.10 V	1.79 V	2.30 V	2.54 V
	**Pt{110}**	2.04 V	1.76 V	2.28 V	2.47 V
	**Pt{100}**	2.05 V (broad)		-	2.42 V
**0.1 M $NaClO_4$/ MeCN**	**Pt{111}**	2.14 V	-	-	2.65 V
	**Pt{110}**	2.22 V	-	-	2.70 V
	**Pt{100}**	2.19 V	-	-	2.66 V

*2D:1S is DMSO: sulfolane (2:1v/v ratio).

Table S3. Corresponding Raman peak positions (cm^{-1}) of 0.1 M $NaClO_4$ in DMSO on different Pt single crystal facets from Figure 3 and Figure 4.

Band Assignment	**Pt(111)**	**Pt(110)**	**Pt(100)**	**Pt(poly)**	**Literature**
$\upsilon_{O\text{-}O}$ **$(O_2^-)_{ads}$**	1107	1105	1115	1115	1109[4] 1108[5]
$\upsilon_{O\text{-}O}$ **$(NaO_2)_{ads}$**	1154	1158	1162	1162	1156 [6, 7]
$\upsilon_{O\text{-}O}$ **(Na_2O_2)**	740<br>798	742<br>798	-	-	736 [4]<br>791 [8]
$\upsilon_{Cl\text{-}O}$ **$(NaClO_4)$**	930	930	930	930	935 [9]
$\upsilon_{CH3(rock)}$ **(DMSO)**<br>$\upsilon_{S=O}$ **(DMSO)**	955<br>1044	955<br>1044	955<br>1044	955<br>1044	1050[10]
$\upsilon_{O\text{-}O}$ **(NaO_2H)**	824	824	-	-	836 [8]
$\upsilon_{O\text{-}O}$ **(H_2O_2)**	874	874	-	-	875[11] 867[12]
$\upsilon_{O\text{-}O}$ **$(HO_2)_{ads}$**	1190	1190	1200	1200	1103[13]*<br>1165[14]

*Limited literature data for presence of HO_2, wavenumber recorded at 14 K under UHV conditions

Table S4. Corresponding peak positions (cm^{-1}) and fitting data of 0.1 M $NaClO_4$ in DMSO on different Pt single crystal facets from Figure S11.

Peak	Peak centre (V vs. Na/Na^+)	Peak Type	Area	% total peak area	FWHM
blue	2.26	Gaussian	31.4	46.2	0.32
red	2.08	Gaussian	4.8	7.1	0.17
black	1.86	Gaussian	32.1	47.2	0.37

References

1. A. Bard and L. Faulkner, *Electrochemical methods: Fundamentals and applications*, John Wiley & sons, Inc., New York, 2001.
2. P. Cao and Y. Sun, *J. Phys. Chem. B*, 2003, **107**, 5818-5824.
3. Z. Deng and D. E. Irish, *J. Phys. Chem. C*, 1994, **98**, 9371-9373.
4. Z. Peng, S. A. Freunberger, L. J. Hardwick, Y. Chen, V. Giordani, F. Barde, P. Novak, D. Graham, J.-M. Tarascon and P. G. Bruce, *Angew. Chem., Int. Ed.*, 2011, **50**, 6351-6355.
5. T. A. Galloway and L. J. Hardwick, *J. Phys. Chem. Lett.*, 2016, **7**, 2119-2124.
6. I. M. Aldous and L. J. Hardwick, *Angew. Chem., Int. Ed.*, 2016, **55**, 8254-8257.
7. N. Ortiz-Vitoriano, T. P. Batcho, D. G. Kwabi, B. Han, N. Pour, K. P. C. Yao, C. V. Thompson and Y. Shao-Horn, *J. Phys. Chem. Lett.*, 2015, **6**, 2636-2643.
8. H. H. Eysel and S. Thym, *Z. Anorg. Allg. Chem.*, 1975, **411**, 97-102.
9. M. I. S. Sastry and S. Singh, *Can. J. Chem.* , 1985, **63**, 1351-1356.
10. W. N. Martens, R. L. Frost, J. Kristof and J. Theo Kloprogge, *J. Raman Spectrosc.*, 2002, **33**, 84-91.
11. S. Venkateswaran, *Nature*, 1931, **127**, 406.
12. P. A. Giguére and T. K. K. Srinivasan, *J. Raman Spec.*, 1974, **2**, 125-132.
13. M. E. Jacox and D. E. Milligan, *J. Mol. Spectrosc.*, 1972, **42**, 495-513.
14. X. Li and A. A. Gewirth, *J. Am. Chem. Soc.*, 2005, **127**, 5252-5260.
